# Biochemical and Cellular Determinants of Renal Glomerular Elasticity

**DOI:** 10.1371/journal.pone.0167924

**Published:** 2016-12-12

**Authors:** Addie E. Embry, Hamid Mohammadi, Xinying Niu, Liping Liu, Borren Moe, William A. Miller-Little, Christopher Y. Lu, Leslie A. Bruggeman, Christopher A. McCulloch, Paul A. Janmey, R. Tyler Miller

**Affiliations:** 1 Nephrology and Medicine, U.T. Southwestern Medical School, Dallas, Texas, United States of America; 2 Center for Matrix Biology, University of Toronto, Toronto, Ontario, Canada; 3 Nephrology, MetroHealth Medical Center, Case-Western Reserve University, Cleveland, Ohio, United States of America; 4 Physiology and Biophysics, Institute for Medicine and Engineering, University of Pennsylvania, Philadelphia, Pennsylvania, United States of America; 5 Medicine, Dallas VAMC, Dallas, Texas, United States of America; Dalhousie University, CANADA

## Abstract

The elastic properties of renal glomeruli and their capillaries permit them to maintain structural integrity in the presence of variable hemodynamic forces. Measured by micro-indentation, glomeruli have an elastic modulus (E, Young’s modulus) of 2.1 kPa, and estimates from glomerular perfusion studies suggest that the E of glomeruli is between 2 and 4 kPa. F-actin depolymerization by latrunculin, inhibition of acto-myosin contractility by blebbistatin, reduction in ATP synthesis, and reduction of the affinity of adhesion proteins by EDTA reduced the glomerular E to 1.26, 1.7, 1.5, and 1.43 kPa, respectively. Actin filament stabilization with jasplakinolide and increasing integrin affinity with Mg^2+^ increased E to 2.65 and 2.87 kPa, respectively. Alterations in glomerular E are reflected in commensurate changes in F/G actin ratios. Disruption of vimentin intermediate filaments by withaferin A reduced E to 0.92 kPa. The E of decellularized glomeruli was 0.74 kPa, indicating that cellular components of glomeruli have dominant effects on their elasticity. The E of glomerular basement membranes measured by magnetic bead displacement was 2.4 kPa. Podocytes and mesangial cells grown on substrates with E values between 3 and 5 kPa had actin fibers and focal adhesions resembling those of podocytes in vivo. Renal ischemia and ischemia-reperfusion reduced the E of glomeruli to 1.58 kPa. These results show that the E of glomeruli is between 2 and 4 kPa. E of the GBM, 2.4 kPa, is consistent with this value, and is supported by the behavior of podocytes and mesangial cells grown on variable stiffness matrices. The podocyte cytoskeleton contributes the major component to the overall E of glomeruli, and a normal E requires ATP synthesis. The reduction in glomerular E following ischemia and in other diseases indicates that reduced glomerular E is a common feature of many forms of glomerular injury and indicative of an abnormal podocyte cytoskeleton.

## Introduction

Mechanical or elastic properties of tissues are a specific, differentiated characteristic that have evolved to adapt glomerular cells to the functions that are required of glomeruli in their unique physical environment. Examples of these adaptations in other tissues include bone that is rigid and skin that is highly elastic and flexible [1]. In renal glomeruli, glomerular capillaries are exposed to relatively high hemodynamic pressures, but have little mechanical support from surrounding tissue. The capillary wall must be able to accommodate blood pressures on the order of 50/40 mm Hg and maintain the structural integrity of the capillary and slit diaphragm [2]. With reductions in renal mass and progression of renal disease, glomerular capillary systolic pressures can increase to 65 mm Hg, and these increased pressures are sufficient to cause capillary injury and glomerulosclerosis [3]. Disease can also arise from abnormal glomerular structure as is the case with mutations in glomerular cytoskeletal, adhesion, basement membrane (GBM), or regulatory proteins, many of which are part of, or interact with the cytoskeleton [4–6]. Mutations in genes that code for mitochondrial proteins can lead to glomerular disease, demonstrating the importance of energy metabolism in the maintenance of glomerular structure [7–9].

**Fig 1:**
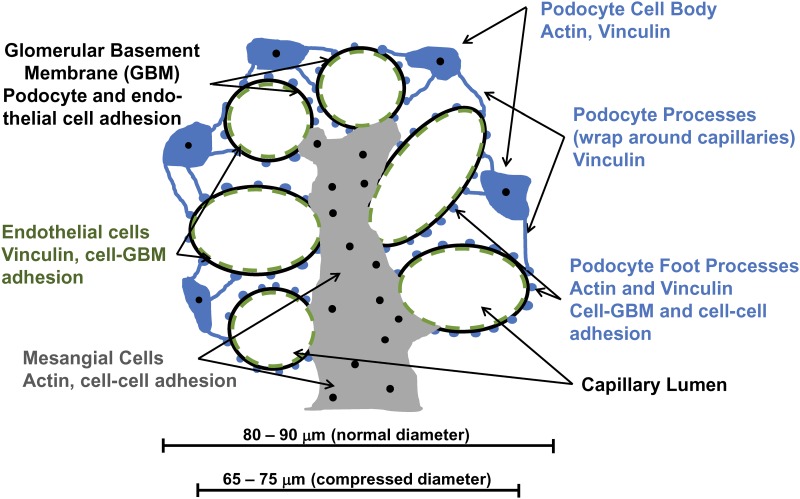
Glomerular structure and summary of findings. The diagram represents a section though the center of a glomerulus showing capillary walls made up of podocyte foot processes, endothelial cells, and the GBM. Podocytes are shown in blue (cell body, processes, and foot process as round structures on the surface of capillary walls attached to the GBM), endothelial cells in green (broken circle inside capillaries signifying fenestrated endothelium attached to the GBM), mesangial cells in grey, and the GBM in black. In reality, but not shown in the diagram, podocyte processes that contain vinculin wrap around the capillaries and appear to provide structural support. Below the label for each structure are listed the cell components affected by the various experimental interventions described in the results section. ATP production is not shown because it is essential to the function of all cellular components. The scale bars below the glomerulus show the approximate size range of glomerululi before (normal diameter, 80–90 μm) and after compression (compressed diameter, 65–75 μm), and indicate that compression (15 μm) by micro-indentation involves capillaries and not the mesangial region of the glomerulus.

The shape, size, and mechanical properties of glomerular capillaries are determined by the behavior of podocytes, the glomerular basement membrane (GBM), and regions of attached mesangium (See summary Fig 1). The capillary wall and GBM are not rigid, but distensible, at least within the range of physiologic and pathophysiologic stresses [4,10]. Endothelial cells have insufficient cytoskeletal structure to provide mechanical support to glomerular capillaries, so podocytes appear to be primarily responsible for the structural integrity of capillary walls [11]. Several studies estimated the elasticity of the glomerular capillary wall, but accurate measurements have not been made in vivo. The factors that contribute to the E of glomerular capillaries are important to understand because they may be modifiable in disease to improve outcomes.

In vivo studies of viscoelastic response are currently not feasible because techniques for simultaneously measuring force in the capillary and displacement of the wall need to be developed. Kriz and co-workers performed studies of increased renal (glomerular) perfusion pressure using uninephrectomy with calculations of renal blood flow, as well as isolated ex vivo perfused kidney models with renal artery pressures likely to be in the physiologic range (65 or 105 mm Hg). In both sets of studies, glomerular capillary diameter was measured in fixed tissues immediately following perfusion, and the authors demonstrated pressure-induced dilation of glomerular capillaries [10,12]. In the uninephrectomy model, they also showed that glomerular capillary dilation was increased by vasodilators, presumably reflecting the effects of increased pressure and flow. Cortes et al perfused isolated glomeruli at pressures ranging from 0 to 160 mm Hg, calculated changes in glomerular volume from measurements of glomerular diameter, and calculated a value for compliance of 2575 μm^3^/mm Hg, units that are not used commonly [13]. As described in more detail in the discussion, depending on the geometry of the model used, these values suggest that distending capillaries over the physiologic range of pressures results in values for the glomerular capillary E of 2.4–4 kPa. More recently, we measured the E of mouse and rat glomeruli using atomic force microscopy, micro-indentation, and microfluidics, and obtained values of 2–3 kPa [14–16]. Although these studies measured isolated glomeruli that were not perfused, they detected physiologically meaningful alterations in glomerular elasticity because they demonstrated a reduced E with actin depolymerization (cytochalasin-D and latrunculin) and inhibition of non-muscle myosin II activity (blebbistatin) as well as in two disease states, Tg26 mice, a model of HIV nephropathy, and Col4a3^-/-^ mice, a model of Alport syndrome [15,16]. The reduced glomerular E in these models was surprising because the conventional view is that following injury, fibrosis develops progressively leading to a stiff, nonfunctional tissue.

In the current work, we define a number of the factors that determine the elastic properties of glomeruli using isolated glomeruli and micro-indentation to measure glomerular E. Elasticity, stiffness, deformability, and compliance, are terms that all describe changes in the shape or volume of substances in repsonse to force. Elastic modulus is a measurement of the deformation or a substance in response to a force. Young’s modulus (E), the simplest case, is force/deformation, and is measured in Pascals (Pa, pressure). Compliance is the inverse of stiffness, and is expressed as 1/Pa. We use E expressed in Pa because this terminology and measurement is used in most of the biophysics literature. We treat intact glomeruli with agents that affect the state of the cytoskeleton and energy metabolism. We measure GBM E directly using magnetic bead displacement, and assess the behavior of cultured podocytes and mesangial cells on variable stiffness matrices that bracket a plausible range of E values for glomeruli. In aggregate, the data indicate that the value for E of glomeruli is 2–4 kPa, and that a relationship exists between the value for E in GBM and glomerular cells growing on it. A normal value for glomerular E is dependent on actin and intermediate filament cytoskeletal structure as well as intact energy metabolism. The behavior of cultured podocytes and mesangial cells on matrices with E values from 5 to 1.8 kPa, supports an E value for the GBM and glomeruli of 2–4 kPa. Finally, we find that renal ischemia and ischemia—reperfusion injury reduce the E of glomeruli consistent with the role of energy metabolism in maintaining the biophysical properties of glomeruli. These results demonstrate that a reduced E is part of glomerular injury by a number of mechanisms, that the podocyte cytoskeleton maintained by active metabolism and adhesion proteins is the primary determinant of the glomerular E, and that elastic environment contributes to the behavior of podocytes and mesangial cells.

## Materials and Methods

### Isolation of glomeruli

Adult mice were sacrificed using CO_2_ narcosis followed by exsanguination. Kidneys were removed from mice, placed in ice-cold 1x PBS+, and dissected. Kidneys were minced with a razor blade and worked through a series of sieves (180μm, 90μm, 45 μm; W.S. Tyler Co., Cleveland, OH) with a rubber plunger from a 5 mL syringe. The post-screen homogenate, which included glomeruli and small tubule fragments, was collected off of the 45um screen and maintained in DMEM with 0.1% BSA. Glomerular aliquots were treated with Latrunculin B (1 μM), Blebbistatin (100 μM), Jasplakinolide (1 μM), Rotenone (1μM), 2-deoxyglucose (2 mM), Sodium Azide (10 μM), Withaferin A (6 μM), EDTA (10 mM), or MgCl_2_ (5 mM) for 2 hours prior to elasticity measurements.

### Glomerular viability assay

Glomeruli were isolated from mice by sieving and washing with 1x PBS. Isolated glomeruli were suspended in RPMI 1640 plus 0.1% BSA and treated with 1μM Latrunculin B (Sigma), 6μM Withaferin A (Fisher Scientific), 2mM 2-deoxyglucose (Sigma), or DMSO for control samples. Treated glomeruli were incubated on ice for 2hrs. Samples were treated with calcein AM and propidium iodide (Life Technologies) for 30 min at 37°C. Drops of each sample were added to slide, covered with coverslips and imaged immediately using fluorescent microscopy at 20x magnification.

### Measurement of glomerular elasticity

The elastic moduli of glomeruli isolated from 3–6 month old mice were measured using and microprobe indentation device (12, 13). Briefly, a tensiometer probe (Kibron, Inc., Helsinki) with a 250 um radius flat-bottomed needle was mounted on a 3-D micromanipulator with 160 nm step size (Eppendorf, Inc.) attached to a Leica DMI 3000B microscope. A glass slide containing a dilute sample of glomeruli was imaged by bright field illumination and the bottom of the probe was brought through the air-water interface until it was just about the surface of a single glomerulus of an approximate 50 μm diameter. The sizes of glomeruli were measured by light microscopy. The probe was calibrated using the known surface tension of a pure water/air interface, and the stress applied to the probe as it was lowered onto the glomerulus was measured as a function of indentation depth. Indentations were kept below 15 μm to avoid large strains that could damage the glomeruli. The deformation geometry is similar to that of a sphere.

### Measurement of filamentous (F) and monomeric (G) actin ratios

Glomeruli were homogenized in buffer (20 mM HEPES pH 7.4, 100 mM NaCl, 1 μM ATP, 1 mM NaVO_4_, 50 mM NaF, 1% Triton X-100, and protease inhibitors) by passage through a 25-gauge needle [16]. The homogenate was centrifuged at 100,000 *g* for 1 h at 4°C. The supernatant (containing G-actin) was removed, and the pellet (containing F-actin) was resuspended in the same volume of buffer containing 15 mM HEPES, pH 7.5, 0.15 mM NaCl, 1% Triton X-100, 1% Na deoxycholate, 0.1% SDS, 10 mM EDTA, 1 mM DTT, 1 mM NaVO_4_, and protease inhibitors. Equal volumes of the fractions containing F and G actin and ~10 mg protein were separated on SDS gels, and the bands corresponding to actin were identified on Western blots with an anti-actin antibody (Cytoskeleton) and standard chemiluminescence. Intensity of staining was quantified from digitized images using NIH Image J software.

### Isolation of glomerular basement membranes

Anesthetized mice were perfused through the left ventricle with PBS containing 4 μm magnetic beds. Glomeruli were isolated as described above, except that in addition, the suspensions of isolated glomeruli were purified further by washing the beads in PBS and then pelleting them with a magnet, removing the supernatant containing tubule fragments, and then resuspending them in PBS. The glomeruli were decellularized by washing them in PBS containing detergents and DNAse [17]. The resultant solution contained single GBMs with two or more beads in capillary lumens.

### Measurement of glomerular basement membrane elasticity

Fragments of GBM aggregates containing magnetic beads (4.5 μm) on the order of 25 x 100 X 150 μm were sandwiched between two 3 x 5 cm coverslips (130 μm thick) that had been sialinized with aminopropyltriethoxysilane (APTES; Sigma A3684, Oakville, Ontario, Canada) and treated with 0.1% w/w glutaraldehyde (GA, Sigma G5882) for 15 min on a shaker and rinsed three times (each time for 5 min) with autoclaved water to prevent movement of the samples. The GBM aggregate fragments with embedded magnetic micro-beads were imaged with a Leica confocal microscope at 63 X using 0.5 μm sections, and visualized from reconstructed *z*-stacks in the imaging mode (XYZ). A magnetic force (~13 nN) was applied for 5 min, and a stack of images around the magnetic bead was acquired before and after application of magnetic force. The magnet was switched off, and the sample was imaged again. The movement of multiple beads in response the magnetic force represents deformation of the GBM aggregate, and was used to calculate its elastic modulus (Young’s Modulus) based on the method of Kamgoue et al [18]

### Immunofluorescence

Differentiated podocytes or mesangial cells were plated on acrylamide gels with variable elasticities overnight and then fixed with 4% paraformaldehyde for 10 min at room temperature. Cells were permeabilized with 0.2% Triton-X-100 in 1X PBS for 30 minutes, washed once, and blocked in 1X PBS with 10% BSA for 1 hour at room temperature. Primary antibody against vinculin (Mouse monoclonal, Santa Cruz) was added at a 1:1000 dilution and incubated overnight at 4 degrees C. The following day, slides were washed and incubated with Alexafluor secondary antibody (Invitrogen) and rhodamine phalloidin (Invitrogen) for 1 hr, washed, and mounted using Prolong Gold with DAPI. Slides were imaged using UV fluorescence on a Leica DMI 3000B microscope at 40X magnification.

### Cell culture

A temperature-sensitive podocyte cell line derived from C57BL/6 WT mice (Leslie Bruggeman) was cultured and maintained at 33°C in RPMI 1640, 10% FBS with 10ug/mL IFN-y. Podocytes were differentiated at 37°C by removing IFN-γ from the medium and culturing for seven days. Similarly, a temperature-sensitive mesangial cell line derived from C57BL/6 WT mice (Leslie Bruggeman) was maintained at 33°C in DMEM/F-12 medium with 1x ITS and 10% FBS. Cells were moved to 37° degrees C for 7 days to differentiate.

### Histology

Mice were anesthetized and perfused with 4% Paraformaldehyde and kidneys removed. Kidneys were fixed in 4% PFA overnight at 4 degrees before submission in 1X PBS to the UT Southwestern Molecular Pathology Core for paraffin embedding, sectioning and Periodic Acid Schiff staining. Sections were imaged using bright field microscopy on a Leica DMI 3000B microscope at 40 X magnification.

### Transmission electron microscopy

Kidneys from anesthetized mice were collected and 1 mm sections were fixed overnight at 4 degrees C in a fresh 2% paraformaldehyde/2% glutaraldehyde solution before submission to the UT Southwestern Electron Microscopy Core Facility for TEM processing. Negative-stained tissues were imaged with a FEI Tecnai G2 Spirit Biotwin transmission electron microscope at 1400x magnification.

### Ischemia models

Ischemia was induced in male C57BL/6 mice 6–8 weeks of age (Jackson Laboratories, Bar Harbor, ME) as described previously (16). Briefly, mice were anesthetized using isoflurane at 37.1°C by a TR-200 system with a rectal probe (Fine Science Tools, Foster City, CA). After a right nephrectomy, the left pedicle was occluded with a micro-aneurysm clip (Codman, Rayham, MA) for 20 min. After 20 min., the kidney was either harvested (ischemia) or the clip was released, the abdomen closed, and the mouse allowed to recover for 45 min. After recovery, the mouse was sacrificed and the kidney collected for glomerular isolation. All experiments followed an approved UT Southwestern IACUC protocol.

### qRT-PCR

Differentiated podocyte and mesangial cell lines were cultured on acrylamide gels of variable stiffness for 24 hours. Cells were harvested and RNA was extracted with a Qiagen RNEasy extraction kit. cDNA was generated using an ABI First Strand synthesis kit (Invitrogen) and qRT-PCR was performed using Sybr Green master mix with an ABI Step One machine (clarify). Gene expression was probed with the following oligos for Filamin-fwd: GATGCACCGTGGAAGAAAAT, rev: GTGGGTCTTTGGTTGTGCTT; Myh9-fwd: GAGCGATACTACTCAGGGCTT, rev: TCTTGCTCTTGTGTGAGGAGG; and WT-1-fwd: TGAAAAGCCCTTCAGCTGTC, rev: GGAGTTTGGTCATGTTTCTCT.

### Acrylamide gel substrate preparation

Variable stiffness polyacrylamide gels were prepared as previously described (34, Pelham and Wang, 1997). Gels were tuned to specific elasticities by varying the concentration of Bis-acrylamide added. Gels were coated with 50mM sulfosuccinimidyl 6 (4’-azido-2’-nitrophenyl-amino) hexanoate (Sulfo-SANPAH, Pierce) in HEPES, pH 8.5 buffer and exposed to UV light for 10 min to photoactivate the crosslinker. Sulfo-SANPAH was removed, washed with HEPES buffer, and incubated with 0.2mg/mL type I collagen (Sigma) solution overnight at 4°C. Prior to use, gels were washed with 1xPBS.

## Results

### Cytoskeletal elements as determinants of glomerular elastic modulus

**Fig 2:**
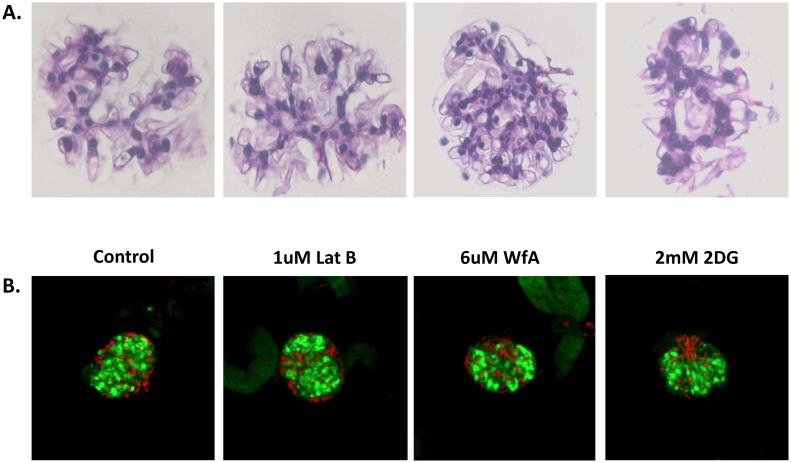
Representative glomeruli isolated by sieving. A. Mouse glomeruli were isolated by sieving, embedded in agarose, sectioned, and stained with PAS. B. Mouse glomeruli were isolated by sieving, treated as indicated, and stained with propidium iodide (red) and calcein AM (green). Glomeruli shown are representative of 10 glomeruli in each group.

**Fig 3:**
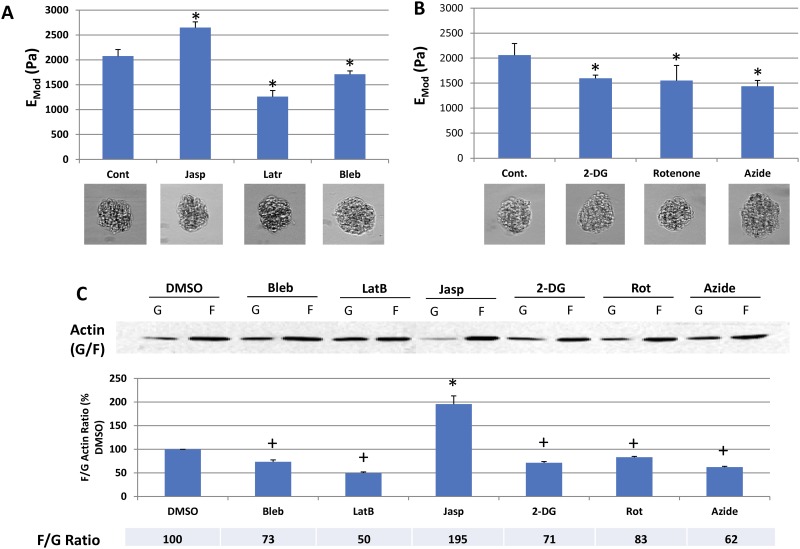
Effect of cytoskeleton-active agents and metabolic inhibitors on glomerular elastic modulus and F/G actin ratios. The top panel (3A) shows the E of isolated mouse glomeruli treated with DMSO, jasplakinolide (1 μM), latrunculin (1 μM), blebbistatin (50 μM). The second panel (3B) shows the E of isolated mouse glomeruli treated with DMSO, 2-deoxyglucose (11. 1 mM), rotenone, (1 μM), and Na azide (10 mM). Each bar in 2A and 2B represents the E mean +/- SD of three separate experiments, in each of which the E of 6–8 glomeruli was measured. The bottom panels 3A and B shows representative phase contrast images of glomeruli from each condition (40 X magnification). The third panel (3C) shows F/G actin ratios in glomeruli treated with DMSO, blebbistatin (50 μM), latrunculin (1 μM), jasplakinolide (1 μM),), 2-deoxyglucose (11.1 mM), rotenone, (1 μM), or Na azide (10 mM). Each bar represents the mean of three to four separate experiments +/- SD and is expressed as the percent of control for each experiment. * p < 0.01 (Paired t test vs control). The top of 3C is a representative western blot showing F and G actin fractions from each condition. + p < 0.02 (Paired t test) vs control.

**Fig 4:**
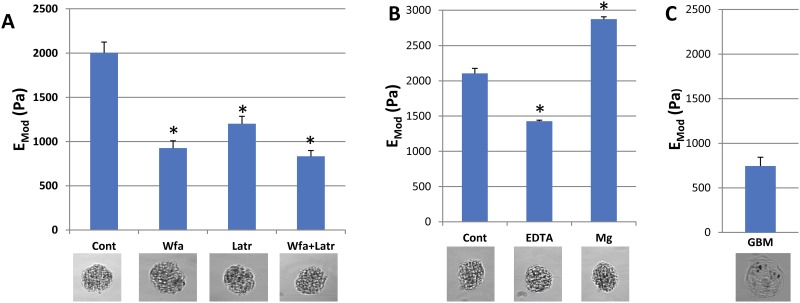
Elastic modulus of glomeruli treated with Wfa and latrunculin, EDTA and Mg, and decellularized glomeruli (GBMs). 4A Isolated glomeruli were treated with Wfa 6 μm, latrunculin 1 μm, or Wfa and latrunculin for 2 hrs in RPMI, and their E values determined by microindentation. Each bar represents the mean of six separate experiments +/- SD, in which four to six glomeruli were measured. In Fig 4B, glomeruli were treated with 10 mM EDTA or 5.0 mM MgCl_2_ for 15 min, and their E was measured using microindentation. Each bar represents the mean value +/- SD from three separate experiments in which 4–5 individual glomeruli were measured. * p < 0.001 (paired t test vs control). 4C shows the mean +/- SD of eight separate measurements of decellularized glomeruli using microindentation. Glomeruli were isolated with magnetic beads, and beads can be seen in the glomerular structures.

The elastic properties of glomeruli could be determined by any of their structural components including the GBM, podocytes, endothelial cells, the cytoskeleton, or adhesion proteins. To determine the contributions of cytoskeletal elements to the overall E of glomeruli, we treated isolated mouse glomeruli (representative examples shown in Fig 2A) for two hours with agents that depolymerize F-actin (1 μM latrunculin), that increase actin polymerization (1 μM jasplakinolide), and that inhibit the activity of non-muscle myosins that are required for mechanosensation and cytoskeletal prestress (100 μM blebbistatin) and measured their E using micro-indentation. To assess viability of glomeruli and be certain that treatment-induced loss of viability did not contribute to changes in the E values for glomeruli, we assessed viability with propidium iodide and caclein staining in control glomeruli and glomeruli treated with latrunculin, withaferin A, and 2-deoxy glucose. Fig 2B shows that although a small number of cells appear dead in all groups, there is no difference in overall glomerular cell viability among the groups that could explain differences in E measurements. Additionally, as shown below (Figs 3 and 4), some treatments increased the value of E, further evidence that the glomeruli are viable, and that treatments do not cause non-specific reductions in E. The calculation for this measurement is based on the first 10–15 μm of compression and should reflect the properties of capillary walls that are on the outside of the glomerulus, and not the central mesangial region (See summary Fig 1). Fig 3A shows that agents that alter the state of F-actin alter the E of glomeruli in a manner consistent with their effects on F-actin. Latrunculin that depolymerizes F-actin reduces the E from 2.1 +/- 0.13 kPa by 40% to 1.26 +/- 0.12 kPa, and jasplakinolide that increases F-actin increases the E by 128% to 2.65 +/- 0.12 kPa. Blebbistatin that inhibits the activity of non-muscle myosins, reduces the E of glomeruli by 28% to 1.7 +/- 0.067 kPa. These results indicate that the state of the actin cytoskeleton is a major determinant of the elastic properties of glomeruli, that the activity of non-muscle myosins that apply stress to the cytoskeleton and that are essential for mechanosensing also make a major contribution.

### Metabolism as a determinant of the glomerular elastic modulus

Maintenance of actin in a polymerized state (F-actin) and the activity of non-muscle myosins both require energy in the form of ATP. To determine the extent to which energy production affects the elastic properties of glomeruli, we treated glomeruli with 2-deoxy glucose (2 mM), a non-metabolizable glucose analogue that blocks glucose metabolism, rotenone (1 μM), that reduces ATP synthesis by inhibiting the transfer of electrons from NADH dehydrogenase (complex I) to ubiquinone, and Na-azide (10 μM), a compound similar to cyanide that inactivates cytochrome oxidase (complex IV). Fig 3B shows that all three compounds lead to a similar reduction in glomerular E from 2.1 +/- 0.2 kPa to approximately 1.5 kPa (2-deoxyglucose 1.53 +/- 0.62 kPa, rotenone, 1.55 +/- 0.30 Pa, and azide, 1.44 +/- 0.11 kPa). Below Fig 3A and 3B are representative images of control glomeruli as well as glomeruli treated with the various agents used in these studies that demonstrate that the overall structure of glomeruli is preserved in the experiments (Also see Fig 2). Fig 3C confirms that the agents that affect the state of actin (F-actin vs G-actin) have the predicted effects on actin polymerization. Latrunculin reduces the level of F-actin by 50 +/- 4.8%, jasplakinolide increases it by 195 +/- 24%, and blebbistatin reduces it by 27 +/- 7.4%. In addition, the agents that interfere with ATP synthesis, 2-deoxy glucose, rotenone, and Na-azide, reduce the levels of F-actin to 71 +/- 5.2%, 83 +/- 3.2% and 62 +/- 3.3% of control, respectively, indicating that the effects of agents that reduce ATP synthesis also reduce the E of glomeruli involves reduction in the level of F-actin in the actin cytoskeleton. These results indicate that maintenance of glomerular structure and mechanical integrity is dependent on the state of the actin cytoskeleton and ATP synthesis, and that inert components of the glomerulus, the GBM, may make a lesser contribution to glomerular mechanics under the conditions of this assay.

### Relative roles of the actin and vimentin cytoskeletons in determining glomerular E

The intermediate filament, vimentin, is primarily expressed in endothelial and vascular smooth muscle cells, but is also highly expressed in podocytes and is the predominant filament in primary and secondary processes [11,19]. To assess the relative roles of the actin cytoskeleton and the vimentin-based intermediate filament cytoskeleton, we treated glomeruli with withaferin A (Wfn, 6 μM), an agent that rearranges intermediate filaments, particularly vimentin, and alters cellular mechanical properties, as well as latrunculin (1 μM) that depolymerizes the actin cytoskeleton [20,21]. As shown in Fig 4A, Wfn decreases the glomerular E from 2 +/- 0.12 kPa to 0.9 +/- 0.08 kPa (55%), a greater effect than latrunculin (1.2 +/- 0.08 kPa). The combination of Wfa and latrunculin reduces the glomerular E to 0.83 +/- 0.07 kPa. Withaferin alters vimentin and other intermediate filament structures so that they condense around the nucleus, producing cells that are more deformable [20,22]. Depending on the cell type, Wfa can also indirectly disrupt the actin cytoskeleton resulting in small aggregates of vimentin and F-actin, an effect that may reflect interaction of the intermediate filament and actin cytoskeletons. A large effect of Wfa on glomerular E is reasonable given the prominence of vimentin structures in podocyte cell bodies and processes. The small degree of additivity with prominent effects of latrunculin and Wfa suggest that the two cytoskeletal systems may interact in podocytes [22,23].

### Role of adhesion proteins in determining glomerular E

To assess the role of adhesion proteins, primarily integrins, in glomerular E, we treated glomeruli with EDTA or Mg and measured their E. EDTA chelates both Ca and Mg, and reduces the affinity of integrins for matrix proteins and cadherins for cadherins on neighboring cells, disrupting both cell-matrix and cell-cell interactions. Exposure of glomeruli to 10 mM EDTA in RPMI for 10 min resulted in a reduction in E from 2.1 kPa to 1.4 kPa (33%) (Fig 4B), comparable to the effect of latrunculin (Figs 3A and 4A). Although the treatment period was relatively short (10 min), EDTA could have additional effects on the cytoskeleton. Mg increases the affinity of integrins for their ligands, but appears not to affect the function of cadherins. Addition of Mg (5.0 mM) in RPMI increases the E of glomeruli to 2.8 kPa, a 140% increase (Fig 4B), slightly greater effect than that of jasplakinolide (Fig 3A). These results indicate that the state of adhesion proteins (low vs high affinity) affects the E of glomeruli to a degree comparable to depolymerization, or maximum polymerization of the actin cytoskeleton.

### Role of glomerular basement membranes in determining E in decellularized glomeruli

To assess the contribution of the GBM to glomerular E using the microindentation assay, glomeruli were isolated from normal mice using magnetic beads, and basement membranes were prepared from the glomeruli using detergent and DNase extraction [17,24]. We measured the E of GBMs using micro-indentation in exactly the same manner as glomeruli, and found that GBMs have an E of 0.74 +/- 0.14 kPa (Fig 4C). This measurement indicates that the major component of the overall E of glomeruli in our assay can be attributed to cellular structures, particularly the cytoskeleton, that also require energy to maintain their mechanical integrity. The similarity of the values for latrunculin + Wfa (0.83 kPa) to the value for decullularized GBMs indicates that the cellular component of the E of glomeruli is primarily determined by elements of the actin and intermediate filament (vimentin) cytoskeletons (Figs 3 and 4).

### Elastic properties of the glomerular basement membrane

Measuring the E of GBMs in a manner that might reflect what a cell would sense is difficult. Ideally, the GBM would be stretched laterally from points that approximate the distance between cell integrin clusters. Although trapping magnetic beads in capillaries would allow deformation of decellularized GBMs with magnetic force (magnetic tweezers), the movement of the capillary loops in relation to each other would be difficult to distinguish from direct stretch of the GBM. Using AFM to deform a GBM would be difficult because it would be impossible to assure uniform orientation of the GBM in relation to the probe. To eliminate the variables of capillary movement and complex 3-D structure, we isolated glomeruli with 4.5 μm magnetic beads, decellularized them, and allowed them to aggregate so that no empty space existed in the aggregates. This preparation contained multiple magnetic beads. The GBM aggregate (~ 30 x 150 x 200 μm) was sandwiched between two glass coverslips treated by APTES and GA, and imaged using a confocal microscope where the location of the beads was defined in three dimensions (X, Y, and Z axes). Following initial localization of the beads, an electromagnet was activated creating a traction force (13 nN) on the beads. After 5 min, the sample was re-imaged with the confocal microscope, and the movement of the beads in response to the magnetic field was determined. At that point, the magnet was switched off, and the relaxation (degree to which the beads returned to their original position) was determined by re-imaging the sample.

**Fig 5:**
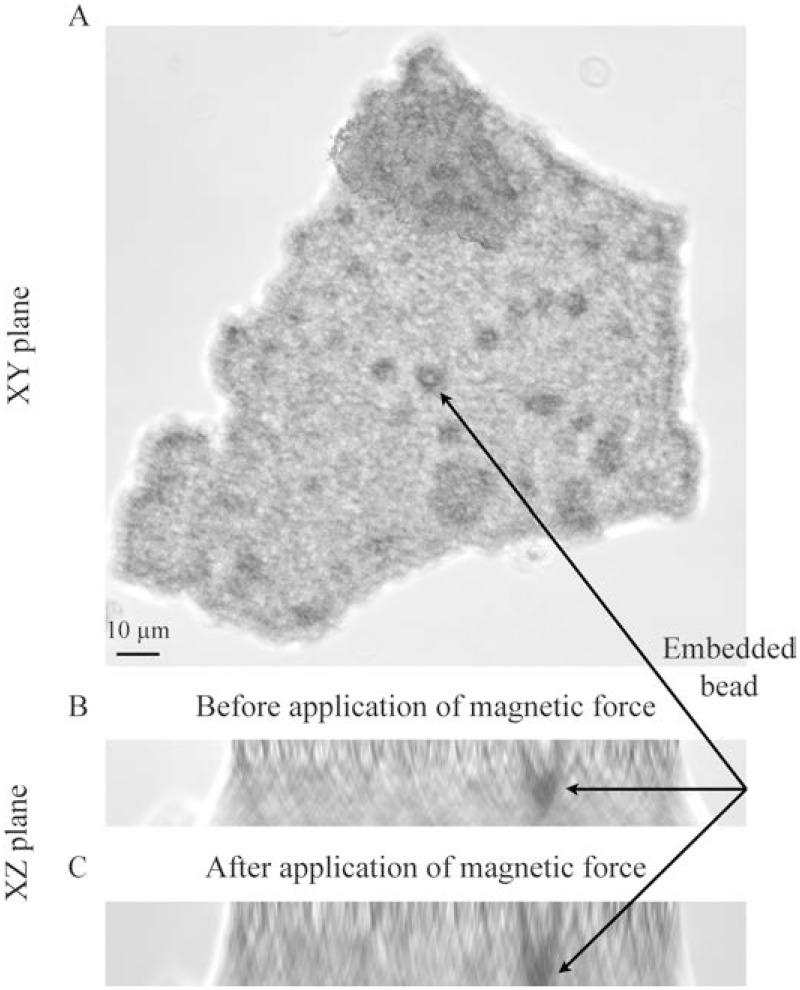
Measurement of GBM E using magnetic bead displacement. Glomeruli were isolated by perfusion with 4.5 μm magnetic beads, and decellularized with detergents and DNase. The GBMs were stored in PBS at 4°C where they formed aggregates. Fragments of the aggregates on the order of 30 x 150 x 200 μm were attached to APTES-coated glass coverslips and imaged using a confocal video microscope (Leica) where the beads seen as dark spots (arrows) were localized in X, Y, and Z dimensions (Panel 5A, XY plane). Panel 5B shows a bead in the XZ plane before activation of the magnet. Panel 5C shows the same bead in the XZ plane 5 min after activation of the magnet. The movement of the bead is evident from its relationship to the bottom of the image. The magnetic force and bead displacement were calculated in the Z axis for four beads, and the Young’s modulus, E, was calculated with the method of Kamgoue et al [18].

Fig 5A shows an image of a GBM aggregate in the XY plane. The dark round objects are magnetic beads. Panel 5B shows the XZ plane before magnetic force is applied, and Panel 5C shows the same bead also in the XZ plane after magnetic force was applied. The movement of the bead is evident from the change in its position relative to the bottom of the panel. Knowing the distance the beads moved in the Z dimension, and the force on the beads in the Z dimension allowed us to calculate a value for E. In each of three samples, we measured the movement of four beads, and they moved 1.34 +/- 0.4 μm in response to an applied magnetic force of 13 nN, resulting in an E of 2.4 +/- 0.73 kPa. With the termination of magnetic force, the beads moved back towards their original position demonstrating elastic behavior of the GBM. We could not calculate elastic vs. viscous behavior because movement of the beads does not correspond to the applied magnetic force in the inelastic regime [25].

This analysis treats the GBM as a solid gel with uniform composition and assumes that the beads (4.5 μm) are sufficiently greater in size than the pores in the matrix fibers, and that movement of the beads represents stretching of the fibers rather than movement of the beads through the pores. It also makes the assumptions that the Poisson ratio of the GBM is 0.5 (like most biologic materials), and that the GBM is purely elastic. The nature of this measurement with the micron scale of bead movement in aggregates that are significantly greater in size, is not sensitive to the direction of deformation, and is likely to be relevant to capillary deformations in vivo in response to cell-generated forces and hemodynamic forces.

### Behavior of glomerular cells on substrates of variable elasticity

**Fig 6:**
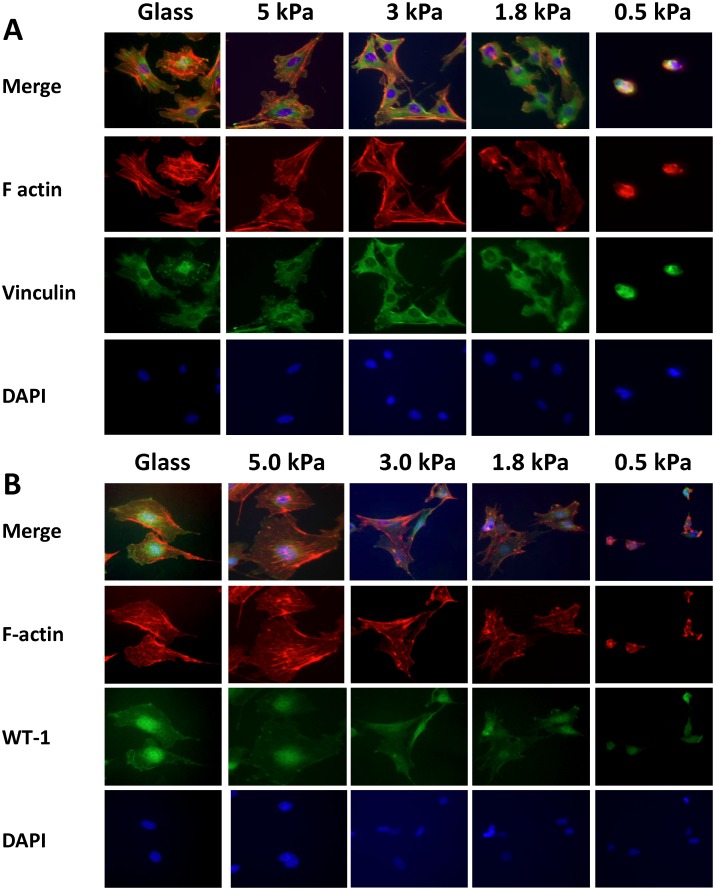
Effects of matrices with different E values on structure and gene expression in cultured mouse podocytes. 6A and 6B show podocytes stained with rhodamine-phalloidin (red) and vinculin (green) (5A) or WT-1 (green) (6B) antibodies, rhodamine-phalloidin (red), and DAPI (nuclear stain) at 40X on glass or PA gels coated with collagen (5 kPa, 3.0 kPa, 1.8 kPa, and 0.5 kPa).

Like other cells, podocytes and mesangial cells respond to their elastic environment, and display differentiated characteristics at values of substrate E that are similar to those found in normal glomeruli. In normal glomeruli, actin fibers and integrin complexes are visible, making the presence of these features characteristic of normal podocytes in vivo [11,19,26]. Based on our work and analysis of data of others, the E for glomeruli should be in the range of 2–4 kPa (see discussion below) [13,14,16]. To evaluate the behavior of podocytes and mesangial cells on substrates ranging in E from rigid (glass, infinitely stiff) to 0.5 kPa (the elastic modulus of fat), differentiated podocytes or mesangial cells were plated on collagen-coated glass (rigid), or collagen-coated polyacrylamide gel substrates of 5.0 kPa, 3.0, kPa 1.8 kPa, and 0.5 kPa, and studied after 24 hrs using immune fluorescence, qRT-PCR to measure levels of different mRNAs, and western blotting [27]. Fig 6 shows spreading of podocytes stained for F-actin (Rhodamine-phalloidin) and vinculin (Fig 6A) or F-actin and WT-1 (Fig 6B) at 40X on the substrates with variable E values. WT-1 (Wilms tumor-1) is a transcription factor that is expressed in, and is characteristic of differentiated podocytes [28]. Podocytes spread maximally on glass, and are small and rounded with minimal visible cytoplasm on 0.5 kPa gels. Podocytes on glass have well-developed stress fibers and mature focal adhesions that appear to be at the ends of stress fibers. Podocytes on 5.0 and 3.0 kPa gels spread slightly less than cells on glass, but maintain identifiable stress fibers and focal adhesions. On 1.8 kPa substrates, the podocytes are less spread, have few identifiable stress fibers as well as diffuse cytoplasmic staining for vinculin and aggregates of vinculin primarily at the edges of cells. These structures may represent immature adhesion structures that co-localize with actin. On 0.5 kPa gels (0.46 +/- 0.064 kPa), the podocytes are small, remain round, and have aggregates of actin and vinculin at the cell periphery without defined structures. These results show that at matrix E values greater than 1.8 kPa, in the 3–5 kPa range, podocytes develop structures characteristic of mature cells in vivo. In a similar series of images, with the same imaging conditions (Fig 6B), WT-1 nuclear staining is visible from glass to 0.5 kPa, and at lower matrix E values, the cells develop a similar appearance to those in Fig 5A with decreased spreading and indistinct structure 0.5 kPa.

**Fig 7:**
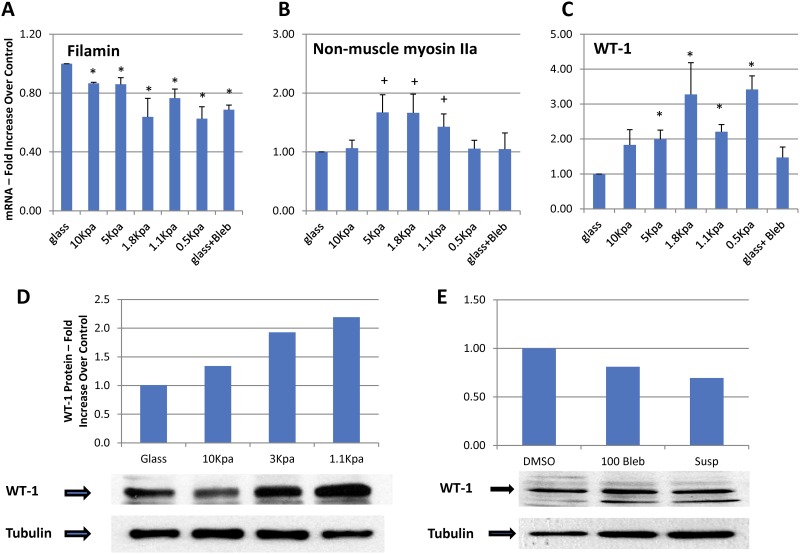
Panels 7A, B, and C show mRNA levels for filamin, non-muscle myosin IIa, and WT-1, respectively, grown on matrices with E values of 10, 5, 1.8, 1.1, and 0.5 kPa, or glass treated with 100 μm blebbistatin, measured by qRT-PCR. Differentiated podocytes were plated on the matrix described and studied 18–24 hrs later. Each bar represents the mean +/- SEM of 4–6 separate experiments. * p < 0.02, + p < 0.05 (paired T test vs control (glass)). 7D shows a representative western blot for WT-1 in podocytes grown on plastic, 10, 3, and 1.1 kPa collagen-coated PA gels. 7E shows a representative western blot for WT-1 from podocytes grown in collagen-coated plastic tissue culture plates, in suspension (bacterial culture plates to which the cells do not adhere), or in collagen-coated plastic tissue culture plates with 100 μm blebbistatin for 18–24 hrs.

Fig 7A–7C show measurement of mRNA coding for three proteins, filamin, non-muscle myosin IIa, and WT-1. Expression levels of these mRNAs change in response to cell matrix elasticity. Filamin mRNA levels increase in some cells in response to stretch, and non-muscle myosin IIa is positively regulated by MRF, a transcription factor in the Rho signaling pathway that responds to the state of the cytoskeleton [29,30]. When differentiated podocytes are plated on substrates of variable E (glass, rigid, through 0.5 kPa) filamin mRNA levels decrease progressively with softer matrix (difference from glass significant beginning at 10 kPa) (Fig 7A). Non-muscle myosin IIa mRNA levels change with the E of the substrate, but with a different pattern from filamin, increasing on the 5.0 to 1.1 kPa gels with a maximum between 5.0 and 1.8 kPa (where the difference from glass is statistically significant), and then returning to a similar level as glass at 0.5 kPa (Fig 7B). WT-1 mRNA levels increase from glass to reach a maximum at 1.8–0.5 kPa (statistically different from glass control from 5 to 0.5 kPa). Fig 7D shows a representative western blot and summary data for WT-1 protein from podocytes grown on glass or gels with E values of 10, 3.0, and 1.1 kPa, and confirms that changes in WT-1 mRNA levels correspond to protein levels with increased WT-1 expression on softer matrix (increase of approximately two-fold from glass to 1.1 kPa). Additionally, when podocytes are treated with blebbistatin (blocks mechanosensation) or grown in suspension (uncoated plastic dishes to which the podocytes do not adhere, Fig 7E), the levels of WT-1 decrease by 20% (blebbistatin) and 35% (suspension). These results indicate that the increased expression of WT-1 by podocytes on soft matrix is a response to matrix elasticity because when mechanosensation is blocked with blebbistatin or the cells are in suspension (no integrin engagement), WT-1 levels decrease. The progressive increase in WT-1 expression with abnormally soft matrix (below 3–5 kPa) is surprising because at these E values the cells appear less well-differentiated, and the reasons for it are not evident. This phenomenon could represent a form of stress response to an abnormal mechanical environment.

**Fig 8:**
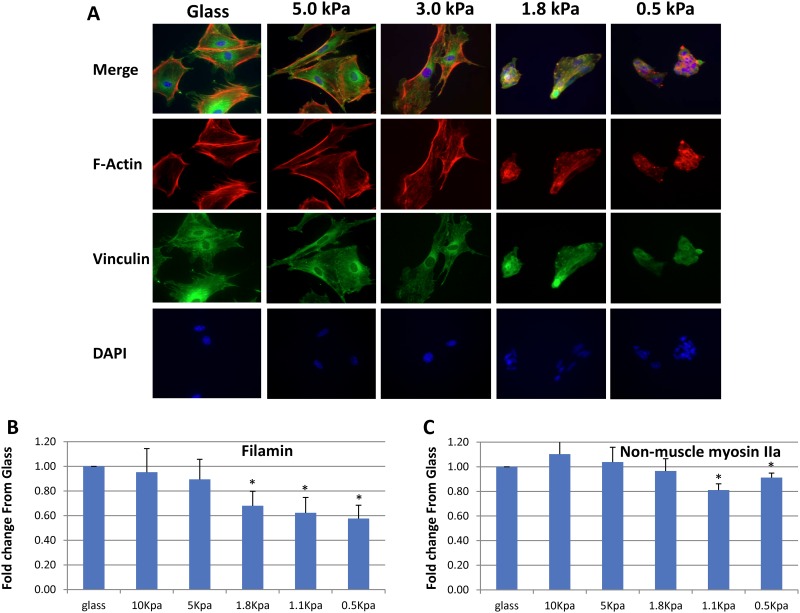
Effects of matrices with different E values on structure and gene expression in cultured mouse mesangial cells. 8A shows mesangial cells stained with rhodamine-phalloidin (red), vinculin antibodies (green), and DAPI (nuclear stain, blue) at 40X on glass or PA gels coated with collagen (5 kPa, 3.0 kPa, 1.8 kPa, and 0.5 kPa). Fig 8B, and 8C show mRNA levels for filamin and non-muscle myosin IIa, respectively, grown on matrices with E values of 10, 5, 1.8, 1.1, and 0.5 kPa, measured by qRT-PCR. Each bar represents the mean +/- SEM of 4–6 separate experiments. *p < 0.05 (paired T test vs control (glass)).

Mesangial cells behave in a manner similar to that of podocytes on the variable elasticity substrates (Fig 8A). Immune fluorescent staining for F-actin (rhodamine-phalloidin) and focal adhesions (vinculin) shows that the cells spread to similar degrees and develop stress fibers and mature focal adhesions on substrates ranging from glass (rigid) to 3 kPa. On 1.8 and 0.5 kPa gels, they do not spread, but form aggregates with indistinct features. Filamin mRNA levels decrease with decreasing substrate E in a manner similar to podocytes (Fig 8B), although there appears to be a step between 5 and 1.8 kPa for filamin where the decrease in mRNA levels is 60–70% that of glass. Although the pattern of non-muscle myosin IIa mRNA expression in mesangial cells appears similar to that in podocytes, the magnitude of increases at matrix E values from 10–1.8 kPa are smaller, and not statistically different from the level of expression on glass (8C). The mRNA levels are statistically less than glass only at 1.1 and 0.5 kPa. These results indicate that although podocytes and mesangial cells respond to their mechanical environment, their patterns of response differ.

### Effects of ischemia on glomerular elastic modulus

**Fig 9:**
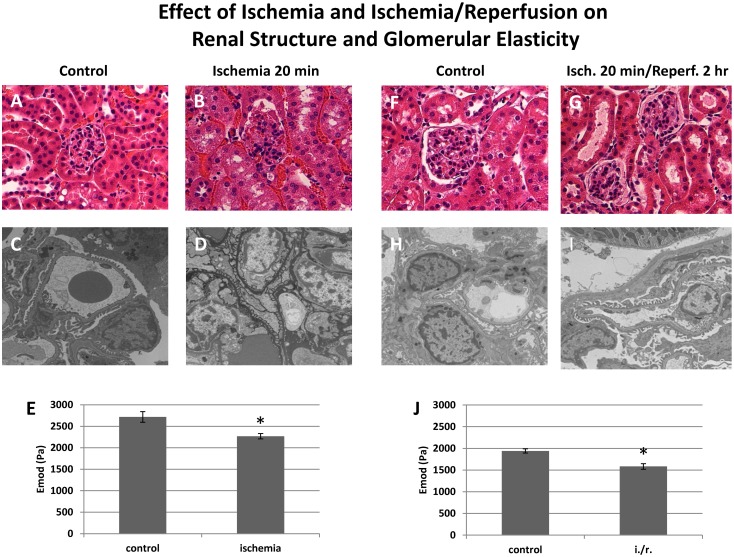
Elastic modulus and structure of glomeruli with ischemia and ischemia-reperfusion injuries. Panels 9A and B show representative H and E stained sections from mouse kidneys featuring glomeruli from control mice (A) and mice with 20 min of ischemia (B) (40 X), and representative transmission electron micrographs of mouse kidney glomeruli from control mice (C) and mice with 20 min of ischemia (D) (1,400 X). 9E shows the E of control mouse glomeruli and glomeruli following 20 min of ischemia. Panels 9F and G show representative H and E stained sections of kidneys following 20 min of ischemia with 2 hr reperfusion (40 X), and 9H and I show representative transmission electron micrographs (1,400 X) from the same kidneys. Panel J shows E or Glomeruli from control kidneys and kidneys following 20 min of ischemia with 2 hrs of reperfusion. Each bar in E and J represents the E +/- SEM derived from separate measurement of 6–8 glomeruli. * P < 0.05 (Paired T test vs control).

The effects of ischemia on glomeruli are not as well studied as those of renal tubular epithelial cells, but work to date indicates that the podocyte cytoskeleton is altered in a manner similar to that in other forms of podocyte injury with effacement of foot processes and rearrangement of the cytoskeleton [31,32]. The data in Fig 3 showing that reduced ATP synthesis leads a reduced glomerular E, indicate that ischemia should lead to reduction in the E of glomeruli in vivo. Unilateral renal ischemia was induced in mouse kidneys by ligation of one renal artery for 20 minutes (9A –E), or 20 minutes of ischemia and I hour or reperfusion (Fig 9F–9J). Control kidneys were harvested at the time the contralateral renal artery was ligated. Following ischemia or ischemia—reperfusion, kidneys were harvested and processed for light and electron microscopy, and the E of glomeruli was measured in glomeruli isolated from the control and ischemic kidneys. By light microscopy, the kidneys injured by ischemia (Fig 9B) or ischemia-reperfusion (Fig 9G) appear relatively normal compared to their respective controls (Fig 9A and 9F). Transmission electron microscopic (TEM) images of glomerular capillary walls in the control kidneys, podocyte foot processes are intact and appear normal (Fig 9C and 9H). However, in the capillaries from the ischemic kidney, the foot processes appear edematous without internal structures and with loss of slit diaphragms (Fig 9D). In the capillary loops from the kidney with ischemia-reperfusion injury, the capillary walls contain regions of intact-appearing foot processes as well as abnormal, effaced foot processes (Fig 9I). Ischemia (9E) and ischemia reperfusion (9J) resulted in similar reductions in E in glomeruli, approximately 20%, similar to that observed with agents that interfere with ATP generation (Fig 3B). These results demonstrate that despite the apparently normal appearance of glomeruli by light microscopy following ischemic injury, they are abnormal structurally and mechanically such that they would be more susceptible to hemodynamic trauma with normal or increased blood flow.

## Discussion

The mechanical properties of glomerular capillaries are a differentiated tissue characteristic that allows them to perform their physiologic functions that include glomerular filtration and opposing hemodynamic forces. To accommodate variable pulsatile flow, glomerular capillaries need to be elastic and not rigid, but with sufficient strength to withstand pressures up to 60 or more mm Hg. Multiple factors determine the elastic properties of glomerular capillaries and glomeruli. These include the GBM, the nature and strength of interactions among cells, and cells and the GBM, the state of the actin cytoskeleton of glomerular cells, and the state of intermediate filaments and microtubules (Summary Fig 1). We focus on the actin cytoskeleton because mutations in a number of genes that affect the actin cytoskeleton cause familial glomerular disease, frequently as a result of podocyte injury.

### Approaches to measurement of the elastic properties of glomeruli

Our studies demonstrate that the mechanical properties of normal glomerular capillaries are primarily determined by their cellular components, and more precisely by energy-dependent cytoskeletal structures (Fig 1). The E of glomeruli is 2.2–2.5 kPa using three different methods, micro-indentation, atomic force microscopy, and a microfluidic method [14,16]. Each of these methods deforms glomeruli by compression and measures a different aspect of glomerular elasticity. AFM deforms the cortical actin of podocytes (approximately 50 nm deformation on the external surface of glomeruli). Microindentation compresses capillaries on opposite sides of a glomerulus approximately 16 μm, or less than two capillary diameters so that the capillary walls should be deformed, and the mesangium should not contribute to the measurement (Fig 1). Microfluidics compresses the glomerulus into a tapered cone and all structures contribute to the measurement [14–16]. Despite the differences in the nature of the measurements, they provide remarkably similar values. Although our measurement, microindentation, deforms the capillaries by external compression rather than dilation from intracapillary pressure as is the case physiologically, these measurements are meaningful physiologically, because values change appropriately with alterations in the cytoskeleton and disease (Fig 9) [15,16].

To estimate an E for glomerular capillaries based on internal distending force, we used data from Cortes et al who perfused rat glomeruli at progressively increasing pressures (0 to 160 mm Hg) and measured increases in glomerular circumference, related these changes to glomerular volume, and calculated the compliance of these glomeruli (1/Pa) [13]. We used the same data and modeled glomeruli either as a 90 μm diameter sphere or as a continuous 10 μm diameter tube (capillary) that would fill a 90 μm sphere (the size of the glomerulus), and calculated values for E for the sphere and capillary models. The sphere model led to an E of 2.3 kPa, and the capillary model led to an E of 3.9 kPa, values that are comparable to those we measure by compression in these studies and past work (Figs 3 and 4) [14–16]. These values are within the range of what is expected measuring the same tissues using different techniques [27]. These results indicate that not only are the values for glomerular E we measure relevant physiologically, they are close to values obtained from distention of glomerular capillaries by forces that approximate those of physiologic hemodynamic force. The similarity of values from compression and indentation suggests that the structural elements of the capillary wall are relatively insensitive to the direction of the force, and that the capillary wall may resemble and elastic tube with an E in the 2–4 kPa range.

A limitation of our microindentation method is that our calculations are based the Hertz model which was developed for soft solids such as gel spheres [14]. Glomeruli are not solid, but are roughly spherical aggregates of capillaries with small amounts of free-flowing liquid surrounding the capillaries (Figs 1 and 2). Consequently, the Hertz model will not provide an accurate measure of the E of a glomerulus, but may provide a minimum value. The value for E obtained with micro-indentation, 2.1 kPa is similar to the value obtained modeling the glomerulus as a sphere from the glomerular perfusion experiments. In any case, even if the micro-indentation values do not represent the true E of glomerular capillary walls in absolute terms, they are meaningful because they reflect changes in the structure of the cytoskeletons of glomerular cells, cell adhesive interactions, and provide a basis for comparing the elastic state of glomerular capillaries under different conditions. Importantly, these changes relate to states of health and disease (Figs 7 and 9) [15,16].

Additional evidence for a glomerular E value of 2–4 kPa comes from the E value of 2.4 kPa we measured for GBMs, and the studies of podocytes and mesangial cells grown on gels with different E values. The E value we measured by AFM for podocytes on the surface of glomeruli, 2.5 kPa, is compatible with cells grown on a matrix, or in this case, GBM with an E of 2.4 kPa. The E of cells grown on collagen matrices usually does not exceed the E of the matrix, and if the cell is capable of stiffening, will often match the E of the matrix [33,34]. The studies of podocytes and mesangial cells cultured on gels of varying elastic moduli further support an E value in the range of 2–4 kPa for glomeruli. In vivo, F-actin is visible at the basal surface of foot processes, and so may be analogous to stress fibers in cultured cells [11]. Vinculin is seen in a similar distribution in a punctate pattern [19]. At matrix E values at or below 1.8 kPa, podocytes do not spread, do not have identifiable stress fibers or focal adhesions, and mesangial cells show a similar pattern of behavior (Figs 6 and 8). On collagen-coated gels with E values greater than 1.8 kPa, podocytes and mesangial cells spread and have visible focal adhesions and stress fibers, characteristics consistent with differentiated podocytes in vivo. These results indicate that the physiologic E value for the matrix on which podocytes and mesangial cells grow is greater than 1.8 kPa, and taking other data into consideration, probably in the 2–4 kPa range.

### Determinants of the elastic properties of glomeruli—cytoskeleton

F-actin is found in bundles on the basal surface of foot processes, but bundles are not seen in the cell body or major processes. The cortical actin cytoskeleton associated with the plasma membrane is composed of a cross-linked F-actin network that provides rigidity to the cell, but this is not visible with immune fluorescence. Actin-based structures contribute directly to the architecture and maintenance of foot process and the slit diaphragm. Using microindentation, we find that to the extent that latrunculin and jasplakinolide depolymerize and polymerize actin, the E of glomeruli can range from 1.26 to 2.65 kPa, and these values probably reflect effects on cortical actin of podocytes. Diseases like HIVAN and familial forms of glomerular disease such as mutations in α-actinin-4 and inverted formin-2 (INF-2) probably affect the podocyte actin cortical cytoskeleton [35,36].

Vimentin fibers make up a substantial portion of the cytoskeleton of podocytes with prominent fibers in the cell body, primary and secondary processes, and foot processes [11,19]. Based on the magnitude of the effect of Wfa on glomerular E, vimentin is likely to have major structural importance in podocytes and glomeruli. In various experimental systems, vimentin is important for cell adhesion and spreading through interactions with actin, filamin, fimbrin, and Itgβ1 trafficking [23,37,38]. Vimentin contributes to the rigidity of a number of cell types, such as mouse embryo fibroblasts and lymphocytes where it primarily affects perinuclear rigidity and organelle localization, while actin contributes to cortical stiffness [22,39]. The precise role of vimentin in glomerular and podocyte biology and mechanical properties is not established, but it may be different from other cells because its distribution and amount are different in podocytes from other cells [19,22,40]. Vimentin KO mice do not have a renal phenotype under normal conditions, but with ¾ reduction in renal mass, die of renal failure that appears to be mediated by abnormal vascular tone attributable to increased endothelin-1 expression and sensitivity [41]. Vimentin levels increase in Col4a3^-/-^ mice by 5 weeks, and in other renal diseases, but the significance of these changes in expression with disease is not clear [42]. Vimentin mutations in familial or sporadic forms of glomerular disease have not been reported.

Endothelial cells contain vimentin and minimal F-actin. Mesangial cells interact with regions of the GBM and glomerular capillaries, contain dense actin stress fibers, and are contractile. The E of the capillary wall measured with AFM and microindentation, then, is likely to depend on the structure of podocytes and the GBM, but probably not endothelial cells to a significant degree. With large deformations, external or internal, mesangial cells could also contribute to the E of glomeruli, but mesangial cells are not likely to contribute to our microindentation measurements (Fig 1).

### ATP metabolism

The state of the actin cytoskeleton is affected directly by the energy state of cells because interfering with ATP generation results in softening of glomeruli and a reduction in F-actin (Fig 3). Although ATP depletion can lead to assembly of small actin polymers, these are short, found in the region of the nucleus, and are unlikely to affect the mechanical properties of cells through effects on cortical actin networks or filament bundles [43]. These fragments might be difficult to detect with the centrifugation and gel system we used. In the ischemia and ischemia-reperfusion studies, the podocyte cytoskeletons were clearly disordered, and the softening of glomeruli we observed was similar in magnitude to glomerular softening in response to agents that deplete ATP (Fig 3). These results indicate that ischemia with ATP depletion has short-term effects on glomerular structure and elasticity that may be relevant clinically.

### Cell-cell and cell—matrix interactions

In glomeruli as well as other tissues such as liver, the cellular components of tissues through attachment to other cells and matrix determine mechanical properties to a greater extent than matrix alone. Decellularized glomeruli and liver slices have greatly reduced Young’s moduli (compression, Fig 4) and shear moduli (liver) [44]. Chelation of extracellular Ca reduces the affinity of integrins for their ligands as well as the affinity of cell-cell adhesions molecules such as cadherins. This treatment of glomeruli resulted in a reduction in E comparable to depolymerization of actin. Conversely, activation of integrins with Mg stiffens glomeruli to 2.9 kPa, a 40% increase. In normal and fibrotic liver, treatment with a disintegrin results in softening of the two liver samples to a similar degree [45]. These results indicate that in addition to the state of the actin and intermediate filament cytoskeletons, the affinity of adhesion proteins for other cells and matrix make significant contributions to the elastic properties of glomeruli as is the case with liver and other tissues [45].

### Matrix elasticity

The mechanical properties and contribution of the GBM to the elastic properties of glomeruli are not defined. Compression of decellularized glomeruli (GBMs), the same assay used for whole glomeruli, shows that they have approximately 40% the E of intact glomeruli. Although this assay allows direct comparison to intact glomeruli, and indicates that the cellular component of glomerular mechanics is dominant, it is not fully informative regarding the material, or visco-elastic properties of a GBM. Normal adult GBM contains type IV collagen (Col4α3–5), laminin 521, nidogen, agrin, and perlecan, along with lesser amounts of other collagens and proteins [46]. Col I networks have been studied in solutions and gels, and the visco-elastic properties defined under a range of conditions including differences in concentration and cross-linking. Although not studied directly, Col IV networks should be more flexible than Col I gels, but the interactions of Col IV alone or with laminins and the other GBM constituents have not been measured in systems that permit detailed analysis of their visco-elastic properties. The E of tubular basement membranes, another basement membrane with type IV collagen was measured using microaspiration, and values of 0.5–1.5 kPa were found, in a similar range as our value for GBM of 2.4 kPa [47].

The geometry and size of GBMs impose practical limitations on measuring their physical properties. We isolated and purified GBMs using magnetic beads, and then allowed the GBMs to aggregate forming solid structures with embedded 4.5 μm magnetic beads. The preparation was exposed to magnetic force inducing movement of the beads and allowing calculation of an elastic modulus. In this preparation, small molecules may be lost, but collagens, laminins, and cross-linkers, the large molecules that are the primary determinants of the elastic properties of matrix and GBMs are retained. The beads are trapped in randomly-oriented capillary loops in an aggregate of GBMs that has essentially become an elastic solid. The size of the beads in relation to the sizes of the fibers means that the beads will stretch the fibers and are unlikely to move between them or change their orientation. Although for our calculations, we assumed that the GBM is purely elastic, like most biologic materials, it probably has viscous properties as well that we were not able to assess. Some of the small molecules that may be lost from the GBM preparation could contribute to its elastic (or visco-elastic) characteristics, but these more sophisticated analyses will require development of new methods. These studies are the first to measure directly the elastic characteristics of GBMs and provide a reasonable estimate, 2.4 kPa, of the E of GBMs.

### Effects of matrix elasticity on glomerular cells

Matrix elastic modulus affects gene expression in podocytes and mesangial cells. WT-1 mRNA and protein expression increase from glass (rigid, infinite E) to 0.5 kPa and decreases response to blebbistatin (blocks mechanosensing) or growth in suspension (absence of integrin engagement). This result was surprising because we expected that WT-1 expression would be maximal in the 2–5 kPa range, the “normal” E for glomeruli. Increasing WT-1 mRNA expression at E values that are clearly below those of normal glomeruli may represent a form of stress response to the abnormal elastic environment. Additionally, the behavior of the genes for filamin-A and non-muscle IIa, genes that respond to the physical state of cells, is affected by the E of the matrix. The pattern is different for podocytes and mesangial cells, indicating that both cell types are mechanosensitive, but have distinct patterns of response. Finally, podocytes and mesangial cells, like other cell types, respond to changes in matrix elasticity and mechanical environment in a coordinated manner that remains to be fully characterized [48–50].

Podocytes have fundamental importance for glomerular disease based on findings that primary injury of podocytes produces progressive glomerular disease. Mutations in the genes that code for proteins that are expressed in podocytes including nephrin, podocin, WT1, α-actinin-4, inverted formin 2 (INF2), TrpC6, MYH9, and phospholipase Cε1, lead to glomerular disease. Pierson’s syndrome, caused by mutations in laminin B2, a matrix protein, is caused by activation of endoplasmic reticulum stress pathways in podocytes, and so ER stress appears to be a primary disease mechanism in podocytes [51]. Finally, the glomerular disease in HIV-associated nephropathy (HIVAN) is primarily a disease of podocytes. Expression of the HIV genome in podocytes results in a disordered cytoskeleton, softening of the cells, and increased susceptibility of the plasma membrane to rupture [52,53]. To the extent that the mechanics of glomeruli from disease models have been studied (HIVAN, Actn4^-/-^, INF2^-/-^, Col4a3^-/-^ (Alport), Col18^-/-^, protamine, ischemia) [15,16] and unpub. Obs. R.T. Miller, P.A. Janmey, and M.R. Pollak), glomeruli and podocytes from models of these diseases have reduced elastic moduli, so that increased deformability of glomeruli appears to be part of glomerular disease. This reduced elastic modulus could permit greater distension of glomerular capillaries with normal blood pressure and flow, resulting in excessive stretch of podocytes and endothelial cells leading to mechanical injury [54]. The effects of reduced glomerular capillary E on GFR are difficult to predict. GFR is controlled by the relative resistances of the afferent and efferent arterioles. If capillary diameter alters shear force from blood flow, alterations in endothelial NO production could affect arteriolar tone and GFR as well as podocyte contractility [55]. With increased capillary diameter, the GBM could be stretched and become thinner reducing the amount of GBM available for protein diffusion in the permeation/diffusion model, and cause increased urinary protein [56]. Alternatively, stretching the GBM could also result in increased density, reduced diffusion of large proteins, and no change in urinary protein. These scenarios do not include alterations in area available for filtration, an important factor in disease that accompanies podocyte injury. The reduced glomerular capillary E could also change the mechanical environment sufficiently to alter the state of differentiation of glomerular cells, particularly podocytes, and alter their patterns of gene expression as shown in Figs 5 and 6 [50,57]. This latter scenario could lead to altered production of matrix, mediators of inflammation or structural proteins, further contributing to abnormal glomerular function.
